# Does switching from oral extended-release methylphenidate to the methylphenidate transdermal system affect health-related quality-of-life and medication satisfaction for children with attention-deficit/hyperactivity disorder?

**DOI:** 10.1186/1753-2000-3-39

**Published:** 2009-12-10

**Authors:** Oscar G Bukstein, L Eugene Arnold, Jeanne M Landgraf, Paul Hodgkins

**Affiliations:** 1 Western Psychiatric Institute and Clinic, University of Pittsburgh School of Medicine, Pittsburgh, Pennsylvania, USA; 2 The Ohio State University, Columbus, Ohio, USA; 3 HealthActCHQ Inc, Cambridge, Massachusetts, USA; 4 Shire Development, Inc, Wayne, Pennsylvania, USA

## Abstract

**Background:**

To evaluate health-related quality of life (HRQL) and medication satisfaction after switching from a stable dose of oral extended-release methylphenidate (ER-MPH) to methylphenidate transdermal system (MTS) via a dose-transition schedule in children with attention-deficit/hyperactivity disorder (ADHD).

**Methods:**

In a 4-week, multisite, open-label study, 171 children (164 in the intent-to-treat [ITT] population) aged 6-12 years diagnosed with ADHD abruptly switched from a stable dose of oral ER-MPH to MTS nominal dosages of 10, 15, 20, and 30 mg using a predefined dose-transition schedule. Subjects remained on the scheduled dose for the first week, after which the dose was then titrated to an optimal effect. The ADHD Impact Module-Children (AIM-C), a disease-specific validated HRQL survey instrument measuring child and family impact, was used to assess the impact of ADHD symptoms on the lives of children and their families at baseline and study endpoint. Satisfaction with MTS use was assessed via a Medication Satisfaction Survey (MSS) at study endpoint. Both the AIM-C and MSS were completed by a caregiver (parent/legally authorized representative). Tolerability was monitored by spontaneous adverse event (AE) reporting.

**Results:**

AIM-C child and family HRQL mean scores were above the median possible score at baseline and were further improved at endpoint across all MTS doses. Similar improvements were noted for behavior, missed doses, worry, and economic impact AIM-C item scores. Overall, 93.8% of caregivers indicated a high level of satisfaction with their child's use of the study medication. The majority of treatment-emergent AEs (> 98%) were mild to moderate in intensity, and the most commonly reported AEs included headache, decreased appetite, insomnia, and abdominal pain. Seven subjects discontinued the study due to intolerable AEs (n = 3) and application site reactions (n = 4).

**Conclusion:**

This study demonstrates that MTS, when carefully titrated to optimal dose, may further improve child and family HRQL, as well as behavioral, medication worry, and economic impact item scores, as measured by the AIM-C in subjects switching to MTS from a stable dose of routinely prescribed oral ER-MPH after a short treatment period. Furthermore, following the abrupt conversion from oral ER-MPH to MTS, the majority of caregivers reported being highly satisfied with MTS as a treatment option for their children with ADHD.

**Trial Registration:**

NCT00151983

## Background

Attention-deficit/hyperactivity disorder (ADHD) is a common psychiatric disorder of childhood, affecting an estimated 7-10% of school-aged children [1-3] and often persists into adolescence and even adulthood [4-7]. Beyond a greater risk for developing mental health comorbidities such as mood and substance use disorders [8-11], children with ADHD struggle with impairment in academic functioning, self-esteem, and interpersonal relationships [12,13]. Families of children with ADHD frequently experience considerable emotional and financial stressors [14,15]. These harmful effects of ADHD on patients and families make it a public health concern and affirm the need for effective treatment [16].

Psychostimulants are recognized as one of the first-line treatments for children with ADHD [1,3,13,17], and methylphenidate (MPH) has been established as an effective agent in reducing ADHD symptoms [18,19]. Accordingly, oral MPH formulations are frequently prescribed medications for this disorder [20]. The methylphenidate transdermal system (MTS; Daytrana^® ^[Shire Pharmaceuticals Ireland Ltd., Dublin, Ireland]), an alternative to oral MPH formulation, is approved by the US Food and Drug Administration (FDA) as part of a comprehensive treatment for ADHD in children aged 6-12 years [21]. MTS is a diffusion-based patch, using DOT Matrix^® ^technology (Noven Pharmaceuticals, Miami, FL), that continuously delivers racemic MPH when applied to intact skin [22]. The dose of drug delivered depends on the surface area of the patch and the length of time that the patch is worn, which allows for tailoring the duration of medication effect with reductions of first-pass metabolism and fluctuating plasma concentrations encountered with oral medications [23,24]. The efficacy and tolerability of MTS have been demonstrated in several clinical studies of school-aged children [25-28].

ADHD treatments, such as MTS, can lead to reduced frequency or severity of symptoms; however, these treatments may not directly translate to improvements in the patient's health-related quality of life (HRQL) at home, at school, or with peers. The behavior rating scales commonly used to assess efficacy are not specifically designed to evaluate the effect of treatment on the everyday lives of children and families living with ADHD. In recent years, the development of multidimensional HRQL questionnaires has afforded clinicians the opportunity to measure the health of children with ADHD more comprehensively [29-37]. Subsequently, a number of reports have been published regarding the improvement of HRQL for children with ADHD receiving pharmacotherapy, primarily atomoxetine, a nonstimulant medication approved by the FDA for the treatment of ADHD [38-46]. To date, there are no published data documenting changes in HRQL after subjects switch from oral ER-MPH to MTS. The purpose of this research was to examine whether changes in HRQL and medication satisfaction occur after switching from a stable oral ER-MPH dose to a carefully titrated, optimized dose of MTS after 4 weeks of treatment.

## Subjects and Methods

### Subjects

Children aged 6-12 years, with a confirmed diagnosed of ADHD (any subtype) by the *Diagnostic and Statistical Manual of Mental Disorders Fourth Edition, Text Revision *(*DSM-IV-TR*^®^, [American Psychiatric Publishing, Inc., Arlington, VA]), and whose parent/legally authorized representative (hereafter referred to as caregiver) was considering a change in treatment based on efficacy, tolerability, or compliance were eligible to participate in the study. Subjects were required to have their ADHD symptoms adequately controlled on a stable dose of oral ER-MPH (Ritalin LA^® ^[Novartis AG, Basel, Switzerland], Concerta^® ^[Alza Corporation, Palo Alto, CA], or Metadate CD^® ^[UCB Inc., Atlanta, GA] not to exceed 54 mg/day) for at least 30 days prior to screening. At baseline, subjects were required to have a total score of ≤ 1.5 standard deviations (SDs) from age-appropriate norms on the ADHD-Rating Scale-IV (ADHD-RS-IV) [27], normal laboratory parameters, vital signs, electrocardiogram (ECG), and a body mass index (BMI) not exceeding the 90^th ^percentile.

Based on medical history collected at screening, children were excluded from study enrollment if they had any comorbid psychiatric diagnosis (with the exception of oppositional defiant disorder), mental retardation, or any concurrent illness or skin disorder that might compromise safety or study assessments. Subjects could not have taken clonidine, atomoxetine, antidepressants, antihypertensives, medications with central nervous system effects, sedatives, antipsychotics, anxiolytics, anticonvulsants, or other investigational medications within 30 days prior to screening.

Data were collected at 18 sites across the United States; the Institutional Review Board at each site or a central Institutional Review Board approved the study. The subject agreed to the study and the subject's caregiver provided written consent in accordance with the International Conference on Harmonisation Good Clinical Practice Guideline E6 and applicable regulations.

### Study design

This was a prospective, open-label, multicenter study of MTS primarily designed to evaluate effectiveness and tolerability after abrupt conversion from ER-MPH to MTS [47]; however, important secondary objectives, HRQL and medication satisfaction, were also evaluated and are the primary focus of this report.

**Figure 1:**
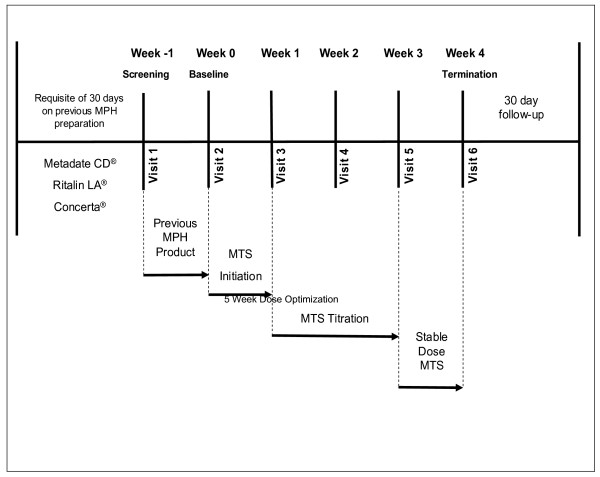
**MTS abrupt conversion study design**. MTS = methylphenidate transdermal system; MPH = methylphenidate.

The study consisted of 4 experimental periods: screening, baseline/MTS initiation, MTS adjustment, and MTS maintenance (Figure 1). In addition, a follow-up phone call was made to caregivers 30 days following the last dose of study drug to access any additional or ongoing adverse events (AEs).

Subjects entered the screening period on their existing dose of oral ER-MPH and continued that medication until the baseline visit at which time they were switched to MTS using a predefined dose-transition schedule (Table 1). Patches were to be applied to alternating hips once daily in the morning and worn for up to 9 hours each day. Subjects remained on their initial MTS transition dose for 1 week and then entered a 2-week dose-adjustment period. Titration to a higher dose or tapering to a lower dose of MTS was permitted based on tolerability and scores on the Clinical Global Impression-Severity (CGI-S) scale. For titration purposes, response to MTS was categorized by the investigator into 1 of 4 conditions and associated actions: "Intolerable" (unacceptable safety profile); "Ineffective" (if the subject's CGI-S score had become worse compared with baseline, or the subject had a CGI-S score of 4 [moderately ill] or worse); "Acceptable" (similar or better ADHD symptom control compared with the baseline ADHD-RS-IV score, with minimal side effects and CGI-S score of 3 [mildly ill] or better); or "Optimal", indicating superior ADHD symptom control (CGI-S score of 2 [borderline mentally ill] or 1 [not ill at all, normal]).

**Table 1:** MTS transition schedule and final dosages by ADHD medication at baseline

Baseline ADHD Medication	Baseline Dose, mg/d	Converted MTS Dose, mg/9 hour	Final MTS Dose Subjects, n (%)			
			10 mg (n = 23)	15 mg (n = 30)	20 mg (n = 52)	30 mg (n = 59)
Concerta^®^ (n = 112)	18 (n = 13)	10	5 (38.5)	5 (38.5)	3 (23.1)	0
	27 (n = 24)	15	1 (4.2)	9 (37.5)	10 (41.7)	4 (16.7)
	36 (n = 46)	20	0	1 (2.2)	26 (56.5)	19 (41.3)
	45 (n = 2)	20	0	0	1 (50.0)	1 (50.0)
	54 (n = 27)	30	0	0	1 (3.7)	26 (96.3)
Ritalin LA^®^ (n = 26)	10 (n = 3)	10	2 (66.7)	1 (33.3)	0	0
	20 (n = 9)	10	4 (44.4)	3 (33.3)	2 (22.2)	0
	30 (n = 9)	15	0	3 (33.3)	5 (55.6)	1 (11.1)
	40 (n = 2)	20	0	1 (50.0)	0	1 (50.0)
	50 (n = 3)	30	0	0	0	3 (100.0)
Metadate CD^®^ (n = 26)	10 (n = 1)	10	1 (100.0)	0	0	0
	15 (n = 1)	10	1 (100.0)	0	0	0
	20 (n = 12)	10	8 (66.7)	4 (33.3)	0	0
	30 (n = 7)	15	1 (14.3)	3 (42.9)	1 (14.3)	2 (28.6)
	40 (n = 3)	20	0	0	2 (66.7)	1 (33.3)
	50 (n = 2)	30	0	0	1 (50.0)	1 (50.0)

MTS = methylphenidate transdermal system; ADHD = attention-deficit/hyperactivity disorder.

"Intolerable" responses required the subject to be tapered to a lower dose, if available. "Ineffective" responses required increasing the subject's MTS dose to the next available strength if side effects permitted. "Acceptable" responses permitted the subject to be maintained on the current dose for the remainder of the dose-optimization phase or, if in the investigator's opinion there was potential for further symptom reduction, try the next higher dose. No further titration was permitted after the final dose-adjustment visit at the end of week 3, and subjects were maintained on their dose of MTS through the final week. Subjects were followed up via phone call to caregivers at 30 ± 3 days after the last dose of study medication to assess any new or ongoing AEs.

### Health-related quality of life and medication satisfaction measures

As secondary objectives of this study, caregiver-reported HRQL and medication satisfaction were assessed using the ADHD Impact Module-Child (AIM-C) and a Medication Satisfaction Survey (MSS).

The AIM-C is a psychometrically sound and well-validated, disease-specific HRQL measure that includes multiple items and scales that assess the impact of ADHD symptoms on children and their families [33]. Previous psychometric data for the AIM-C instrument has indicated excellent item convergent and discriminant validity as well as very strong reliability with Cronbach *α *scores of 0.88 and 0.92 for the child and family subscales, respectively. No floor effects and limited ceiling effects (2%) were observed in a previous sample [33].

The AIM-C consists of 2 core scales to assess child (8 items) and family (10 items) HRQL. The measure also consists of 2 multi-item scales to assess medication tension/worry (3 items regarding tension in administering and taking ADHD medication) and missed-doses worry (4 items regarding worry about the number of daily doses missed at home or school). In addition, the AIM-C includes 10 clinical treatment questions (including a missed-dose item and 5 items that ask about behavioral change and management), a 6-item school cooperation scale, 9 parent attribute/knowledge items, 4 economic impact items, and 4 demographic questions answered by the subject's caregiver. Not all items and scales were utilized in the study (refer to the Appendix 1). The AIM-C was completed by the subject's caregiver at baseline and at week 4.

The MSS is a nonvalidated 11-question survey designed to assess caregiver satisfaction with the efficacy and tolerability of MTS. The questions were answered by the subject's caregiver at week 4. Responses ranged from "strongly agree", "agree", "somewhat agree", "strongly disagree", "disagree", and "somewhat disagree". For MSS data analysis, "strongly agree", "agree", and "somewhat agree" were combined into the category "agreed". "Strongly disagree", "disagree", and "somewhat disagree" were combined into the category "disagreed".

### Tolerability measures

Tolerability was based on spontaneous reports of AEs. Any AEs were coded using the *Medicinal Dictionary for Regulatory Activities *(MedDRA) Version 7.0. Reported AEs were monitored and recorded throughout the study and for 30 days after the last dose of study drug. Investigators categorized AE intensity as "mild", "moderate", or "severe".

Tolerability has been briefly discussed herein to provide context to those MSS items relating to the known side effect of stimulant medications, including MTS, as rated by caregivers.

### Statistical analysis

Subjects who received at least 1 MTS patch application and who had at least 1 ADHD-RS-IV measurement on or after week 1 were included in the intent-to-treat (ITT) population. Analyses of HRQL and MSS data were conducted for the entire ITT population as well as the following ITT population subgroups: prior treatment (Concerta, Ritalin LA, and Metadate CD), age (6-9 year olds and 10-12 year olds), and gender.

Scores for the AIM-C Child Impact Scale, Family Impact Scale, Medication Tension/Worry Scale, and Missed-Doses Worry Scale were computed separately by summing the items within each scale and deriving an overall mean score. The raw mean scores for all 4 scales were then transformed on a 0-100 continuum, with higher scores indicating less negative impact or improved HRQL. Thus, increasing AIM-C scores for these 4 scales is indicative of improvement. The 5 AIM-C behavior items, the 1 missed-dose item, and the 4 economic impact items were treated as discrete categorical variables.

Summary statistics were presented for the 2 primary HRQL AIM-C scales, Child and Family Impact, as absolute change from baseline or number and percentage of subjects experiencing outcomes. In the case of absolute change from baseline, descriptive (number, mean, SD, median, minimum, and maximum) statistics were computed. Findings for the remaining secondary portions of the AIM-C, including the 2 worry scales, 5 behavior items, the missed-dose item, and the 4 economic impact items, were briefly summarized using descriptive statistics.

Data for MSS were summarized as number and percentage of subjects with the following responses to each of the survey questions using descriptive statistics: "strongly agree", "agree", "somewhat agree", "strongly disagree", "disagree", and "somewhat disagree".

The safety population was defined as all subjects who received at least 1 MTS patch application. Safety-related information was evaluated using descriptive statistics. Adverse events were considered to be treatment emergent if they began on or after the first patch application, or on or before 30 days after the final patch application in this study.

## Results

### Subjects

**Figure 2:**
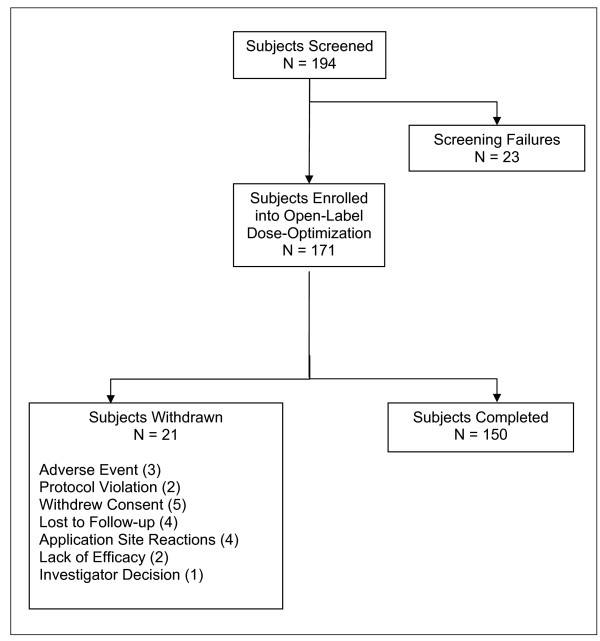
**Subject disposition flow chart**.

A total of 171 subjects were enrolled in the study. Subject demographics and baseline characteristics are shown in Table 2. All subjects received at least 1 MTS patch application; therefore, all subjects were included in the safety population. The ITT population was made up of 164 subjects; each received at least 1 MTS patch and had at least 1 ADHD-RS-IV evaluation on or after week 1. Three subjects were discontinued from the study due to AEs resulting in 150 subjects (87.7%) completing the 4-week study (Figure 2).

**Table 2:** Subject demographics and baseline characteristics, ITT population

Characteristic, N = 164	Baseline	Endpoint
Age, mean (SD), years	9.4 (1.9)	-
Sex (%)		
Boys, n	117 (71.3)	-
Girls, n	47 (28.7)	-
Race, n (%)		
White	129 (78.7)	-
African American	19 (11.6)	-
Asian	1 (0.6)	-
Native Hawaiian/ Other Pacific Islander	1 (0.6)	-
American Indian/ Alaska Native	1 (0.6)	-
Other	13 (7.9)	-
Weight, mean (SD), lb	75.6 (26.0)	-
Height, mean (SD), in	54.2 (5.2)	-
ADHD-RS-IV Score (SD)		
Total	14.1 (7.5)	9.9 (7.5)
Inattentive	7.9 (4.3)	6.4 (5.3)
Hyperactive/impulsive	6.2 (4.5)	4.4 (4.5)
AIM-C Child Impact Score (SD)^a^	62.4 (20.5)	73.4 (18.4)
AIM-C Family Impact Score (SD)^a^	68.2 (23.3)	78.4 (21.7)

^a^Based on 161 subjects

ITT = intent to treat; ADHD-RS-IV = ADHD-Rating Scale-IV; AIM-C = ADHD Impact Module-Child

### Health-related quality of life

#### AIM-C Child Impact Scale

Across all MTS doses, child HRQL scores had a tendency to change for the better rather than to deteriorate. At baseline, while subjects were receiving a stable dose of oral ER-MPH, the AIM-C child HRQL mean (SD) score was 62.4 (20.5). At end of study, the child HRQL mean (SD) score increased to 73.4 (18.4), reflective of a 17.5% increase after 4 weeks of treatment with MTS.

Subgroup analyses conducted by prior treatment with Concerta, Ritalin LA, and Metadate CD revealed that changes in mean from baseline to the end of study in the AIM-C child HRQL score were 10.3, 17.1, and 7.3, respectively. Those subjects previously taking Ritalin LA experienced the largest percentage increase (28.7%) in child HRQL scores from baseline to end of study.

Improvements in child HRQL from baseline to end of study were also noted in both the 6- to 9-year-old and 10- to 12-year-old age groups; however, changes in mean AIM-C child HRQL scores from baseline were greater for subjects aged 6-9 years (12.3) than those aged 10-12 years (9.6). Gender analysis revealed a greater improvement in mean scores on the child HRQL measure from baseline to end of study for girls (14.9) compared with boys (9.4).

#### AIM-C Family Impact Scale

Average family HRQL improved from baseline to end of study for all MTS dose groups. At baseline the family HRQL score (mean [SD]) was 68.2 (23.3). Family HRQL mean (SD) score at end of study was 78.4 (21.7), reflective of a mean absolute change from baseline of 10.2 and a mean percent improvement of 15.0% in family HRQL.

Subgroup analyses by prior treatment revealed similar changes in mean from baseline scores for Concerta (9.7), Ritalin LA (9.2), and Metadate CD (13.0). For subjects aged 6-9 years, the change in mean family HRQL scores from baseline to end of study (14.4) was more than double that demonstrated by subjects aged 10-12 years (6.1), reflective of a 23.5% improvement in family HRQL after 4 weeks of MTS treatment. Gender analysis revealed a greater improvement in child HRQL mean scores from baseline to end of study for girls (11.8) versus boys (9.5).

#### AIM-C behavior Items

AIM-C items regarding behavioral issues were generally improved over the course of the study. From baseline to endpoint, percentages of caregivers agreeing that their child's behavior "stayed the same" (ie, did not fluctuate throughout the day as before) increased (35.4-73.3%), those agreeing that their child's behavior tended to be up and down at different times throughout the day decreased (64.0-24.8%), and those agreeing that their child's behavior became more problematic at night decreased (68.3-39.1%). At study endpoint the majority of caregivers (85.1%) reported that handling changes in their child's behavior was not very difficult or a little difficult, and 63.8% reported that they were "very often" or "fairly often" successful in getting their child to focus or regain self-control.

Subgroup analyses based on prior treatment, age, and gender demonstrated similar trends in these AIM-C behavioral items.

#### AIM-C overall worry scales

Overall improvements from baseline to endpoint were seen in the Medication Tension/Worry Scale and School/Missed-Doses Worry Scale. On the Medication Tension/Worry Scale, the mean (SD) score at baseline was 68.0 (24.95). At endpoint, the mean (SD) score was 85.4 (17.4), indicative of a change in mean from baseline of 17.3 and a percent improvement from baseline of 25.4%.

Subgroup analyses revealed that improvement in mean scores from baseline to endpoint with regard to the Medication Tension/Worry Scale scores were similar for all prior treatment groups (16.7 [Concerta], 16.3 [Ritalin LA], and 20.8 [Metadate CD]) as well as for the 6- to 9-year-old and 10- to 12-year-old age groups (19.0 and 15.8, respectively). Gender analysis demonstrated greater improvement from baseline to endpoint in Medication Tension/Worry Scale scores for girls (22.3) compared with boys (15.4).

On the School/Missed-Doses Worry Scale, the mean (SD) score at baseline was 79.1 (17.8). At endpoint, the mean (SD) score was 90.1 (14.1), reflecting a percent improvement in mean scores from baseline of 13.9%. Subjects that were receiving Metadate CD prior to MTS dose transition showed a greater improvement from baseline to endpoint in the School/Missed-Doses Worry Scale score (15.4) compared with those that were receiving Concerta (10.5) or Ritalin LA (8.7), and improvement for girls (16.3) was nearly double the improvement noted for boys (8.9). No apparent difference was noted between age groups.

#### AIM-C missed-dose item

The percentage of subjects with missed doses was reduced at study endpoint (23.6%) compared with baseline (28.6%), indicating a slight improvement in compliance. This trend was also noted for prior treatment, age, and gender subgroups as well.

#### AIM-C economic items

Overall, there were improvements from baseline to endpoint in all economic impact items. From baseline to endpoint, the percentage of subjects missing school days decreased from 14.9-7.5%. The percentage of subjects requiring no extra hours of tutoring, nursing, home healthcare, or other services increased from 60.2% at baseline to 77.6% at endpoint. Furthermore, from baseline to endpoint, the percentage of caregivers reporting no missed days of work to manage ADHD-related issues increased from 78.3-86.3%. The percentage of subjects with emergency room visits related to their ADHD symptoms was unchanged between baseline and endpoint at 1.9%. Prior treatment, age, and gender subgroup analyses for all AIM-C economic impact items revealed similar results.

#### Medication Satisfaction Survey

Ratings on the MSS are displayed in Table 3. At study endpoint, 93.8% of caregivers were satisfied with their child's use of study medication, and satisfaction levels were high across all patch sizes. Caregiver satisfaction was high regarding MTS administration frequency and ease of compliance. At endpoint, 97.5%, 96.2%, and 97.5% of caregivers were satisfied with how many times a day their child needed to take MTS, how easy it was for their child to take MTS, and agreed that MTS doses were rarely skipped or missed, respectively.

**Table 3:** Caregiver MTS treatment satisfaction at endpoint, ITT population

MSS Summary	Agreement, %^a^ N = 161
Satisfied with frequency of daily dosing	97.5
Satisfied with ease of use	96.2
Child rarely missing a dose	97.5
Satisfied with child's behavior	93.8
Improvement in child's social interactions	82.6
Satisfied with how child pays attention	91.9
Satisfied with duration of effect on symptoms	93.1
Overall satisfaction	93.8

^a^"Strongly agree", "agree", and "somewhat agree" were combined into the "agreed" category.

MTS = methylphenidate transdermal system; ITT = intent to treat, MSS = Medication Satisfaction Survey.

Caregiver satisfaction with the effect of MTS on behavioral symptoms was high (93.8%), reflecting respondents' satisfaction with their child's behavior while he or she received the study medication. The majority of caregivers (82.6%) reported improvements in their child's social interactions since beginning the study medication, and 91.9% were satisfied with the efficacy of the study medication on their child's attention. The majority of caregivers (93.1%) indicated a high level of satisfaction with the duration of effect of the study medication on their child's ADHD symptoms.

Regarding medication satisfaction with tolerability of MTS, at study endpoint the majority of caregivers (59.6%) agreed that the study medication decreased their child's appetite. The majority of caregivers (88.1%) disagreed that the study medication made their child feel sleepy during the day, but 34.8% of respondents agreed that MTS treatment made it hard for their child to fall asleep at night. The number of caregivers reporting concerns about their child's falling asleep at night appeared to be dose related: 5 (33.3%) for subjects receiving MTS 15 mg, 15 (37.5%) for subjects receiving MTS 20 mg, and 38 (34.8%) for subjects receiving MTS 30 mg.

Prior treatment, age, and gender subgroup analyses produced similar results for all 11 MSS items.

### Tolerability

A total of 201 treatment-emergent AEs (TEAEs) were reported by 97 subjects (57%) in the safety population during the 4-week study. Most TEAEs (> 98%) were considered to be mild to moderate in intensity and the most common included headache (5.1%), decreased appetite (4.7%), insomnia (3.5%), and upper abdominal pain (2.7%).

A total of 7 subjects discontinued the study due to intolerable effects. Four subjects withdrew from the study because of reactions at the application site that ranged from definite erythema with severe itching to normal appearance at the patch application site with moderate itching. None of these 4 subjects reported continuing AEs at follow-up. Three subjects reported AEs that led to study withdrawal. Two subjects reported 4 serious AEs that at follow up were reported to have resolved. A 7-year-old boy experienced "worsening of ADHD symptoms" and "condition aggravated" after receiving MTS 10 mg for 1 day; both events were considered unrelated to study treatment. A 12-year-old girl experienced "acute depression" and "suicide attempt" while receiving MTS 30 mg for 16 days; both events were considered possibly related to study treatment. The last subject, a 6-year-old girl, continued to experience anger/irritability after MTS study medication was discontinued.

No deaths were reported during the study. At follow-up, no subject reported continuing AEs.

## Discussion

Overall, AIM-C scores for child and family impact, behavior, worry, missed dose, and economic impact had a tendency to improve with all doses of MTS treatment from baseline to endpoint. Such results are consistent with other published reports of pharmacotherapy impact on HRQL in children with ADHD [38-46]. Interestingly, at baseline, while subjects were taking a stable dose of oral ER-MPH, AIM-C child and family HRQL scores were already above the median possible score. However, AIM-C child and family HRQL scores were further increased at endpoint after 4 weeks of treatment with MTS. These results demonstrate that HRQL changes for both the child and the family living with ADHD can be observed after switching from a stable dose of oral ER-MPH to MTS and carefully titrating to an optimal MTS dose in a relatively short treatment period. HRQL in both children and families improved during this study, which is consistent with behavioral reciprocation often observed in treatment strategies. Although clinical intervention tends to stress the importance of caregivers as change agents of the child, in turn, children are reciprocally change agents of the caregiver and of family life in general.

Caregivers of children in the Ritalin LA subgroup, children aged 6-9 years, and girls reported greater improvements from baseline to endpoint in child HRQL when compared to the other subgroups. Children aged 6-9 years and girls experienced greater improvements from baseline to endpoint in family HRQL when compared with children aged 10-12 years and boys. Improvements seen in family HRQL for the prior treatment subgroups (Concerta, Ritalin LA, and Metadate CD) were not very much different from one another.

Behavior, as measured by the AIM-C, was also generally improved over the course of the study. At baseline, most caregivers indicated inconsistency in their child's behavior across the day in contrast to endpoint where the majority reported consistency in behavior. Further, at study endpoint the majority of caregivers reported that handling changes in their child's behavior was "not very difficult" or only "a little difficult" and that they were "very often" or "fairly often" successful in getting their child to focus or regain self-control. It may be inferred that improvements in behavior and the ability of the caregiver to manage their child's behavior after treatment with MTS may positively impact the HRQL for children with ADHD and their families.

Overall improvements from baseline to endpoint were seen in the Tension/Worry Scale and School/Missed-Doses Worry Scale, with the latter possibly due to a slight reduction in number of missed doses reported by caregivers at study endpoint. Improvement in compliance may reflect increasing capacity to establish a routine of patch administration with time, although noncompliance is often underestimated in any clinical trial.

Improvements from baseline were also noted for the AIM-C economic impact items. Although the majority of caregivers reported no missed school days due to ADHD symptoms at both baseline and endpoint, the number of children who missed 1 or more days of school was lower at endpoint compared to baseline. In addition at endpoint, there was a decrease in the number of missed days from work for caregivers and in the number of subjects requiring extra hours for tutoring, nursing, and home healthcare. The number of subject visits to the emergency room due to ADHD remained relatively unchanged from baseline to endpoint. Although an economic evaluation was not performed, the improvements in HRQL observed in children with ADHD coupled with the decreased direct and indirect costs associated with the disorder may lead to MTS being a cost-effective treatment option from a societal perspective.

Overall, MSS scores were high across all dose groups. When asked to rate their overall satisfaction, caregivers indicated that they were satisfied with MTS as a treatment for their child's ADHD (ie, overall satisfaction, 93.8%; satisfaction with ease of use, 96.2%; and satisfaction with duration of effect, 93.1%). Similar percentages of caregivers were satisfied with the behavioral efficacy of MTS as well. Consistent with the commonly reported TEAEs of decreased appetite and insomnia, the majority of caregivers agreed that the medication caused their child to want to eat less, and approximately one-third of caregivers agreed that the medication made it hard for their child to fall asleep at night. A recently published study evaluating MTS wear times of 4 and 6 hours, instead of the 9-hour wear time used in this study, suggests that some late day side effects may be attenuated by early removal of the patch [25].

### Limitations

The current study was open-label, with no control group, and was nonrandomized. The sample was restricted to subjects who were previously taking a stable dose of oral ER-MPH with ADHD symptoms that were within 1.5 SDs above age/sex norms. Thus, these results may not be generalizable to subjects who were not taking a stable dose of oral ER-MPH or who had either a poor or excellent response to oral ER-MPH prior to switching to MTS.

All study subjects were abruptly switched to MTS from a stable dose of their previous oral ER-MPH. Motivation for participation in this study may have been prompted by low efficacy, high prevalence of AEs, or low HRQL with subjects' previous medications. These motivations for study participation may have contributed to a bias toward HRQL improvement.

HRQL was assessed using the AIM-C, a validated caregiver-completed measure. As noted earlier, the AIM-C consists of a 10-item scale to assess the impact of ADHD on the caregiver and life at home. In addition, the caregiver is also asked to report on how well their child is completing chores/homework, behaving in public, and working to his or her potential. The AIM-C Child Impact Scale also includes items such as how comfortable the child seems with others, how well the child seems to get along with others, and how well he or she handles everyday hassles. Thus, caregiver ratings in these latter areas (although still valid from a personal perspective) provide a more indirect account of the everyday impact of ADHD on the HRQL of the child.

During the study, management of subjects involved regular visits, dose adjustments, and careful attention to AEs. This optimal management may also have had a role in improving behavior, HRQL, and satisfaction.

Subjects who failed to respond to psychostimulants in the past, and those with conduct disorder, were excluded from the study. Therefore, the results of this study should not be extrapolated to these patient populations.

The current study was of a relatively short duration and was designed to evaluate subject response after abrupt conversion from ER-MPH to the MTS dose specified by the dose-transition schedule. Thus, the safety data presented herein should not be considered representative of the long-term safety of MTS or the safety of MTS in treatment-naïve children with ADHD. Also, although a predefined dose-transition schedule was utilized in this current study, because of differences in bioavailability between products, MTS dose should be titrated based on individual response when patients are switched from oral ER-MPH formulations.

Despite these limitations, the results of this study imply that MTS may further improve child and family HRQL, as well as behavior, medication worry, and economic impact item scores, as measured by the AIM-C in subjects switching from a stable dose of oral ER-MPH over a short treatment period. Furthermore, following the abrupt conversion from oral ER-MPH to MTS, the majority of caregivers were highly satisfied with MTS as a treatment option for their children with ADHD. Further studies evaluating the long-term impact of MTS on HRQL as well as analysis of probable predictors of HRQL change and/or satisfaction will be important in understanding what elements of stimulant treatment contribute most to improvements in HRQL and satisfaction. These studies will require additional controls on medication administration, including optimal dosing for the compared agents, as well as the use of blinded evaluators, and a validated medication satisfaction instrument.

## Conclusion

This study demonstrates that MTS was well tolerated and, when carefully titrated, may further improve child and family HRQL, as well as behavioral, medication worry, and economic impact item scores, as measured by the AIM-C in subjects switching to MTS from a stable dose of oral ER-MPH after a short treatment period. Furthermore, following the abrupt conversion from oral ER-MPH to MTS, the majority of caregivers were highly satisfied with MTS as a treatment option for their children with ADHD.

## Competing interests

OGB receives or has received research support and/or acted as a consultant for Ortho-McNeil Pharmaceuticals, Problem-Based Learning Institute, Quintiles CME, Routledge Press, and Shire Pharmaceuticals. LEA receives or has received research support, acted as a consultant, and/or served on a speaker's bureau for Autism Speaks, Neuropharm, NIMH, Novartis, Organon, Reach Institute, Ackerman Foundation, Shire Pharmaceuticals, and Targacept. PH is an employee of Shire Pharmaceuticals. JML is vice president and chief scientific officer with HealthActCHQ, which owns the intellectual property rights to the AIM-C survey.

## Authors' contributions

All authors have made substantial contribution to the conception, design, and/or conduct of the study, have been involved in the drafting and/or critical review of this manuscript, and all authors have given final approval of this manuscript.

## Appendix 1

### ADHD Impact Module - Child (AIM-C)^©^

#### Item Content

Thinking about your child's ADHD during the past 2 weeks, how much does each statement describe your child?

• My child did well following through with chores and homework on his/her own

• My child seemed to get along with kids his/her age

• Feedback from teachers and parents about my child with ADHD has been generally positive

• My child was able to deal well with everyday hassles and frustrations with friends and siblings

• My child behaved well in public places like stores/restaurants

• My child seemed comfortable with how things are going with friends and siblings

• My child worked to his/her potential

• My child adapted well to disruptions or unexpected changes in his/her routine

In general, how closely do each of the following statements describe life for you and your family?

• My child's ADHD has added stress to our home life

• My child's ADHD limits me from entertaining at home

• I avoid going certain places or doing certain things with my child because of problems with his/her ADHD

• I feel as if I am "on guard" in public settings because I never know if my child with ADHD will act up

• My child's ADHD limits what we can do as a family

• I am anxious about my child's future because of the ADHD problems he/she is having

• I feel tired and worn out because caring for a child with ADHD requires a lot of my time and energy

• I feel my child's ADHD controls my life

• I am frustrated that my child's ADHD is unmanageable

• I am concerned about the long-term effect my child's ADHD behavior may have on his/her siblings

Please tell us how long your child has been on the following treatments for his/her ADHD.

*(NOTE: not used in the current study but part of the AIM-C)

Which of the following statements best describes your child's current ADHD medication status?

* *(NOTE: not used in the current study but part of the AIM-C)

Are the following statements TRUE or FALSE for your child?

• My child's behavior stays about the same throughout the day

• My child's behavior seems to be up and down at different times throughout the day

• My child's behavior becomes more problematic at night

• Is it difficult to handle changes in your child's behavior when the medication wears off?

In general, how often are you successful in getting him/her to focus or regain self-control?

How would you rate the school's cooperation in each of the following areas?*

*(NOTE: this scale was not used in current study but is part of the AIM-C)

• Diagnostic/testing process for ADHD

• Obtaining additional academic services

• Developing a classroom behavior management plan

• Designing a homework "plan of action"

• Giving your child his/her ADHD medicine

• Ongoing support in managing your child's ADHD

Please answer each of the following questions:

• How much tension is there between you and your child about taking his/her ADHD medication?

• How much do you worry about your child missing a dose of ADHD medication at school or when you are not around?

• How bothered are you by having to monitor your child's ADHD medication everyday?

• How embarrassed do you think your child is about taking ADHD medication at school?

• How much does your child resist taking medication at school?

• How often does your child's dose at school get missed or forgotten?

• How often has your child had problems because of a missed dose at school?

During the last 7 days, how many scheduled doses of your child's ADHD medication have been missed?

How strongly do you agree or disagree with the following statements?*

*(NOTE: Not used on this study but included in the AIM-C)

• ADHD can be helped by drugs alone

• ADHD can be helped by behavioral interventions and therapy/counseling WITHOUT medication

• ADHD can be helped by behavioral interventions and therapy/counseling with medication

• ADHD can't really be helped. It's something my child will "outgrow"

During the past 6 months, has your child missed days from school due to problems with his/her ADHD?

During the past 6 months, have you, your spouse or partner missed days from work due to problems with your child's ADHD?

During the past 6 months, has your child required any hours of extra tutoring, nursing, home healthcare, or other services as a result of his/her ADHD?

During the past 6 months, has your child visited an emergency room due to an accident or incident that you believe was related to his/her ADHD?

[Reproduced with permission from HealthActCHQ Inc.^© ^Copyright 2006-2008. All rights reserved.]
